# The Hippo Signaling Pathway as a Drug Target in Familial Dilated Cardiomyopathy

**DOI:** 10.53941/ijddp.v1i1.189

**Published:** 2022-12-21

**Authors:** Paulina Langa, Beata M. Wolska, R. John Solaro

**Affiliations:** 1 Department of Physiology and Biophysics and the Center for Cardiovascular Research,University of Illinois at Chicago, Chicago, IL,USA.; 2 Department of Medicine, Division of Cardiology, College of Medicine, University of Illinois at Chicago, Chicago, IL, USA.

**Keywords:** mutant sarcomere proteins, coronary vasculature, endothelium, fibroblasts, extra-cellular matrix

## Abstract

We focus here on the Hippo pathway in the hierarchical sensing and modulation of the mechanical state of the adult heart in health and disease. The Hippo pathway interrogates the micro-environment of cardiac myocytes providing surveillance of the mechanical state with engagement of signaling pathways critical to homeostasis of cardiac development, remodeling, and function and vulnerable to pathologies. Our discussion centers on Hippo signaling in the altered mechanical state instigated by variants of genes expressing mutant sarcomere proteins that trigger a progression to dilated cardiomyopathy (familial DCM). There is an unmet need for therapies in DCM. Recent progress in the discovery of small molecules that target Hippo signaling and are intended for use in cardiac disorders provides leads for modifying Hippo in DCM. As we emphasize, identifying useful targets in DCM requires in depth understanding of cell specific Hippo signaling in the cardiac micro-environment.

## 1. Introduction: Mechano-Sensing in Familial Dilated Cardiomyopathy

Recent literature provides evidence in support of the relative importance of mechano-sensing and mechano-transduction in adaptive and maladaptive mechanisms in the heart. Stresses and strains are sensed and transduced as biochemical signals. As a reflection of the distinctive coupling of mechanics and function in the heart, the microenvironment of cardiac myocytes possesses an elaborate and complex set of mechano-sensors and transducers. These elements in mechano-transduction within myocytes consist of the array of sarcomere proteins, the cytoskeleton, costamere, dystrophin-complex, microtubules, integrins, and intercalated disks coupled to membrane channels and receptors, the extracellular matrix (ECM), and neighboring cells. Elements in mechano-transduction beyond the myocyte mechanical landscape are present in smooth muscle cells, endothelium and pericytes in the coronary vasculature, fibroblasts, neuronal cells, and adipocytes [1].

As is typical of regulatory mechanisms, there is a window (“sweet spot”) of mechano-transduction activity that maintains myocardial homeostasis; higher or lower activities beyond this homeostatic window may lead to pathology. To stay within the homeostatic window, the heart responds not only to neurohumoral and chemical regulation, but also to regulation within its micro-environment consisting of autocrine, paracrine and mechano-signaling via sensors of the mechanical state, which we discuss here.

Familial cardiomyopathies linked to variants in genes expressing sarcomere proteins provide an example where a dominant biophysical perturbation in mechanical state moves regulatory mechanisms away from the “sweet spot” and triggers a progression to hypertrophic cardiomyopathy (HCM) or familial dilated cardiac myopathy (DCM). Commonly, HCM related mutant sarcomere proteins, especially mutations in thin filament proteins, induce a persistent increase in myofilament response to Ca^2+^ with hypercontractile state, whereas DCM mutants induce a persistent decrease in Ca^2+^ sensitivity with hypo-contractility. Our hypothesis has been that that early interventions that depress Ca^2+^ sensitivity in HCM can prevent the progression of the cardiomyopathies. In the case of HCM, we reported data in support of this hypothesis by creating double transgenic mice expressing the HCM-linked mutant alpha-tropomyosin (αTm) E180G in the cardiac myocyte compartment together with expression of pseudo-phosphorylated cardiac troponin I (S23D and S24D; TnI-PP), which desensitized myofilaments to Ca^2+^ [2]. These studies provided proof of principle for the successful development and clinical use of the small molecule, Camzyos^®^ or Mavacampten, to treat advanced HCM by directly affecting sarcomere activation [3].

We have focused here on DCM. Our rationale is that DCM is a highly prevalent form of heart failure, the most common indication for cardiac transplants, and a common cause of cardiomyopathies in the pediatric population [4]. Familial causes have been identified in ~30 % of the cases with variants in genes expressing titin as the leading cause. A connection between mechano-sensing and the cellular phenotype of DCM has been documented [5]. Moreover, our investigation into the effects of normalization of myofilament response to Ca^2+^ early in DCM showed promise but had limitations [6]. We employed co-expression of slow skeletal troponin I (ssTnI), which sensitized myofilament response to Ca^2+^ in the myocytes of hearts expressing the Ca^2+^-desensitized DCM mutant, αTmE54K [6]. Compared to controls there was a significant prevention of the DCM phenotype in the double transgenic mice. However, as we noted in the published work expression of ssTnI as a mechanism of preventing DCM may not represent a specific modification of myofilament Ca^2+^-sensitivity. We have shown in previous studies that expression of ssTnI in the adult murine heart leads to metabolic remodeling, altered Ca^2+^ fluxes, resistance to acidosis, and altered length dependent activation, the basis of the Frank-Starling relation [7, 8]. Moreover, expression of ssTnI in the DCM mouse model did not have uniform effects on wall thickness, and diastolic function [6]. Whether specific Ca^2+^-activators such as Omecamtiv Mecarbil, which we have shown to increase Ca^2+^ sensitivity in myofilaments regulated by DCM mutant αTmE54K, are effective in treatment of DCM remains an open question [9,10].

## 2. Hippo and Yap Signaling in DCM

In considering the approaches to modify the altered mechanical state early in DCM, we discuss here, the Hippo signaling pathway. In the context of the micro-environment, the Hippo pathway is an overarching signaling cascade in the hierarchy of mechano-sensing. A minimal definition of DCM is the presence of dilated left ventricle contractile dysfunction without abnormal loading. The contractile dysfunction is associated with decreased sarcomere force generating capacity, cell lengthening, energetic dysfunction, fibrosis, coronary vascular compartment disorders, and sudden cardiac death. These abnormalities in the geometry and stiffness of the myocardium alter mechano-signaling and are expected to alter Hippo pathway signaling indicating a possible role of therapeutic approaches modifying this cascade.

**Figure 1.**
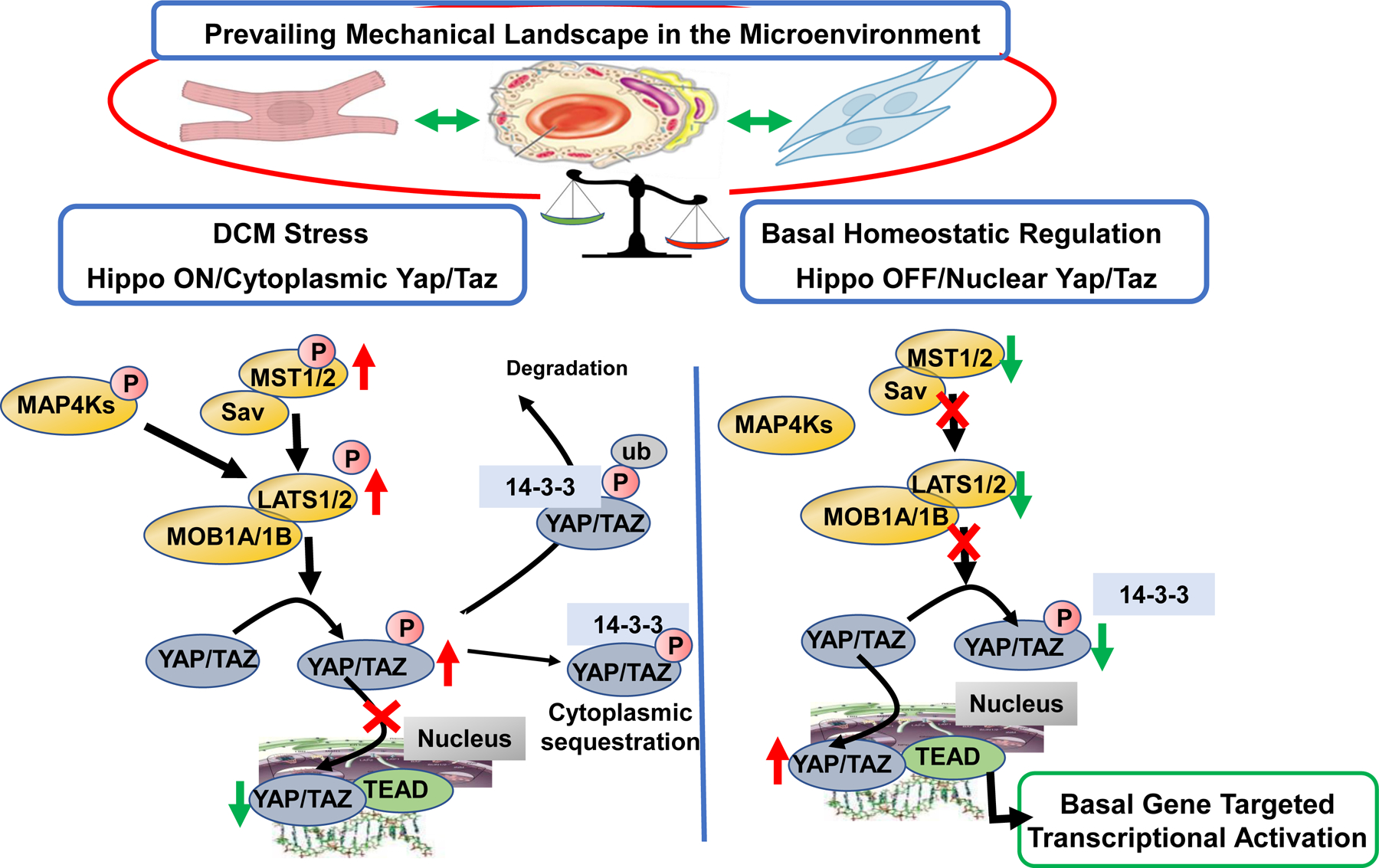
Scheme illustrating OFF states of Canonical Hippo signaling in a basal physiological state in the cardiac micro-environment and in an ON state promoted by familial DCM linked to sarcomere protein mutations. Evidence summarized in the text demonstrates that in the basal homeostatic state there is relative physiological suppression or restraint of phosphorylations in the Hippo pathway, which permits the nuclear transcriptional activator complex Yap (Yes associated protein) and Taz (transcriptional coactivator with PDZ-binding motif) to move from a cytoplasmic location into the nucleus relieving transcriptional repression by TEAD (targeting transcriptional enhanced associate domains). This transcriptional activation is important in homeostasis of cardiac function and structure, vascular and endothelial stability, and homeostatic stability of the extracellular matrix. Evidence indicates that in DCM, there is a shift to the Hippo “ON” or unrestrained state in which Yap is retained in the cytoplasm in a process that depends on a cascade of upstream phosphorylation signals shown in Figure 1. Activation of Mst1/2 (sterile 20-like kinase 1 and 2) in combination with Sav (scaffolding protein Salvador) results in activation of Lats1/2 (large tumor suppressor 1 and 2) that in combination with MOB1A and 1B (Mps one binder 1A and B) promote phosphorylation of Yap/Taz, preventing its entry into the nucleus. As indicated in Figure 1, the cytoplasmic Yap may sequestrate in the cytoplasm, or be removed by ubiquitin related mechanisms involving specific forms of 14-3-3 proteins. As discussed, small molecules inhibiting Mst1 and Lats enzymes have been developed and serve as leads for further development of agents restoring physiological balance in Hippo signaling.

Figure 1 illustrates a scheme summarizing the mechano-signals and their transduction in engaging the core canonical Hippo-Yap signaling pathway in mammals with emphasis on the idea that this mechanism of surveillance of the micro-environment mechanical state is cell specific. Non-canonical pathways leading to Hippo-independent Yap signaling have also been elucidated and are discussed below [11,12]. Excellent reviews provide detailed descriptions of the Hippo pathway that has essential elements, which have remained unchanged throughout evolution [13 – 16]. Hippo signaling is required for homeostatic development of the heart. Although generally considered to be silenced in the adult heart, there is evidence that the pathway is significantly involved in cardiac homeostasis in the adult and activated with cardiac stress. The scheme in Figure 1 emphasizes the role of Hippo signaling in the adult heart. The physiological suppression or restraint of phosphorylations in the pathway permits the nuclear transcriptional activator complex, Yap (Yes-associated protein) and Taz (transcriptional coactivator with PDZ-binding motif) to move from a cytoplasmic location into the nucleus relieving transcriptional repression by TEAD (targeting transcriptional enhanced associate domains). This transcriptional activation is important in development, cell death and proliferation and tissue homeostasis. In the Hippo “on” or unrestrained state, Yap is retained in the cytoplasm in a process that depends on a cascade of upstream phosphorylation signals shown in Figure 1. With activation of Mst1/2 (sterile 20-like kinase 1 and 2) in combination with Sav (scaffolding protein Salvador), there is activation of Lats1/2 (large tumor suppressor 1 and 2) that in combination with MOB1A and 1B (Mps one binder 1A and B) promote phosphorylation of Yap/Taz, disallowing entry into the nucleus. As indicated in Figure 1, the cytoplasmic Yap may be removed by ubiquitin related mechanisms involving specific forms of 14-3-3 proteins [17]. In the Hippo “off” or restrained state, this cascade of reactions is repressed, there is a dominance of dephosphorylated Yap, which enters the nucleus acting as a co-transcriptional regulator disinhibiting TEAD.

Although there is general focus on the canonical Hippo pathway and Yap signaling, there are reports of members of the Hippo pathway that affect cardiac structure and function independent of Yap signaling in the myocardium. Examples of the non-canonical signaling pathway summarized by Del Re are RASSF1A (Ras association domain family 1 isoform) and Mst1 in cardiomyocytes [11]. Not only do up and down regulation of these signaling molecules affect fibrosis, apoptosis, and left ventricular dilation, but Mst1 is a kinase demonstrated to phosphorylate cardiac troponin I with the potential of altering cardiac contractility [18]. This highlights a need to study the potential role of non-canonical pathways also in DCM [12,19–21].

Insights into the transcriptional activation of TEAD have been provided by studies in which there was a conditional ablation of TEAD in cardiac myocytes [22]. These studies revealed that mice with the deletion demonstrated acute lethal DCM associated with loss of transcription of SERCA2a and protein phosphatase-1 (PP1) leading to a decrease in phosphorylated phospholamban (PLN). Analysis of the TEAD global transcriptome also revealed alterations in mitochondrial and sarcomere related pathways. Significant decreases in sarcomere protein messages occurred in the case of cMyBP-C3 (cardiac myosin binding protein C), titin, MHC (myosin heavy chain) α and β, MLC (myosin light chain) 2 and 3, and skeletal muscle actin. These findings provide valuable information on the importance of role of the Hippo pathway in homeostasis of the adult heart and indicate its relevance as a point of vulnerability in DCM.

The importance of Hippo signaling in the adult heart is also indicated by data reported in determination of expression and localization of Yap1, Taz, and Tead1 in samples of human hearts in late stages of stress induced by ischemic injury and idiopathic DCM [23]. The evidence indicated a modification in Yap/Taz signaling in the diseased hearts with a decrease in phosphorylated Yap1 in relation to Yap1 in the nuclear fraction. Compared to controls, the dysfunctional hearts CARP, Ctgf (connective tissue growth factor) and Cyr61 (cysteine-rich angiogenic inducer 61). Studies with a desmin related cardiomyopathy in a mouse model supported the conclusion that Yap/Taz is activated in cardiac stresses. The finding of increased levels of Cyr61 and Ankrd1, both of which are matrix proteins promoting angiogenesis, indicates an involvement of the cardiac micro-environment in Hippo signaling in the human context. Whether these changes were related to adaptations or maladaptations remains unclear as does the modification in the early stages of the disorders.

Wu et al. also provided evidence of a connection of Hippo signaling with a DCM phenotype [24]. DCM was induced in a mouse model by overexpressing Mst1. The overexpression of Mst1 induced a presumably compensatory increase in total Yap expression but with decreased localization of Yap in the nucleus associated with an increase in the ratio of Yap-P/Yap and evidence of a decrease in co-activation of TEAD1. Early onset mechanical dysfunction occurred at 3 weeks of age and continued into adulthood at 6 months of age. Transcriptional modifications in the cardiac myocytes targeted mainly mitochondrial genes. Mitochondria were structurally modified and increased in number. Documented dysfunction in the mitochondrial metabolism was associated with a depression in cellular levels of ATP, elevated lactate, and evidence of oxidative stress. Induction of a DCM phenotype by modifications in Hippo pathway was also reported by Yamamoto et al. [20]

Hippo signaling also plays a role in homeostasis of the extracellular matrix, fibrosis, and the vascular endothelium. Using the same model of Mst1 overexpression as described above, Nguyen et. al showed that by 3–8 months of age there was a ~40 fold increase in expression of galectin-3 (Gal-3), a pro-fibrotic factor, in association with a DCM phenotype with depressed contractility, upregulated fibrotic genes, and significantly increased LV collagen content [25]. Studies by Xiao et al. demonstrated that the basal resting state of cardiac fibroblasts is maintained by Lats1/2 [26]. Deletion of Lats induced a sustained fibrosis. Coronary blood flow was altered in a desmin null mouse model of DCM, but there were no determinants of a role for Hippo signaling [27].

The significance of the coronary endothelium in heart failure, especially in non-ischemic HF such as DCM has been emphasized in a perspective reported by Heusch [28]. Along these lines Hippo signaling has been demonstrated in angiogenesis and endothelial function [14, 29]. It was demonstrated that angiomotin (Amot) restricts Yap and Taz from entering nucleus by promoting sequestration in the cytoplasm in vitro in HUVEC (human umbilical vein endothelial cells) [21]. An interesting observation was done by Ragni et al. [30] who showed that Angiomotin-like 1 (Amotl1) upon interaction with its partner, Fat4, a cadherin, can act as a inhibitor or a facilitator of Yap, and thus affecting thickness of the ventricular walls, further pointing out possibilities for studying the mechanism in cardiovascular diseases, including familial DCM.

Taken together these findings of studies related to mechanisms in DCM indicate that Hippo signaling is a homeostatic mechanism that operates in a basal state in the adult to maintain physiological levels of cardiac myocyte energy generating mechanisms related to mitochondrial function and blood flow, and energy consuming mechanisms in the SR and sarcomeres and maintenance of a stabile mechanical environment via the ECM (Figure 1). Data indicate that when the Hippo pathway is restrained by genetic approaches promoting phosphorylation and cytoplasmic retention of Yap, there is a failure in these homeostatic mechanisms leading to early onset DCM.

## 3. Therapeutic Targeting of Yap and Hippo Signaling

Evidence described above indicates that therapeutic targeting that modifies Hippo signaling by promoting Yap shuttling into the nucleus may be useful in DCM. Kastan et al. reported discovery of a non-toxic Yap activator [31]. The small molecule termed TRULI1 inhibited Lats kinases thereby promoting the dephosphorylation of Yap and increased expression of Yap targets. When added to neonatal rat ventricular myocytes (NRVM) there was an increase in proliferation. Another approach to dephosphorylation of Yap is to target Mst1/2. By specifically blocking the Mst1 ATPase activity with the small molecule XMU-MP-1, Fan et al. demonstrated this compound as a possible facilitator of liver regeneration [32]. This compound provided the first lead for targeted regenerative therapies.

## 4. Conclusions and Perspectives

Evidence cited here supports the potential for developing agents targeting the hippo pathway to meet the need for innovative and effective therapy in a prevalent cardiac disorder. Lead compounds in development are likely to provoke further investigation of Hippo drug targeting in the heart, but also demand in depth understanding of the pathway in specific cells in the micro-environment. Although there are extensive and illuminating series of investigations of Hippo signaling in the heart, these studies have employed cardiac myocyte specific up and down regulation of the pathway [20, 33, 34]. There are few studies integrating the potential effects of the Hippo pathway in the myocyte micro-environment. Del Re has also emphasized that therapeutic targeting of the Hippo pathway demands in depth understanding of Hippo signaling in different cell types in the cardiac myocyte micro-environment [35]. Included is a discussion of the need for definitive understanding of Hippo pathway function in cardiac non-myocytes highlighting the necessity of considering cell type specificity for effective therapeutic targeting of the Hippo pathway in cardiovascular disease. Moreover, evidence that Hippo pathway intermediates such as Mst1/2 may modulate Yap signaling independently of the Hippo cascade emphasizes the need for consideration of the non-canonical Hippo signaling in therapeutic interventions [11]. An approach to address this gap in our understanding is to investigate readouts of up and down regulation of Hippo signaling with determination of cell specific transcription employing translating ribosome affinity purification (TRAP) technologies *in vivo* [36]. As we have previously discussed, these measurements can be combined with proteomic determinations of proteins in the polyribosome/mRNA complexes in cardiac myocytes and various cell types in the micro-environment

Development of small molecules modifying the Hippo pathway intended for therapy in cancer provide important information related to the development of agents for use in cardiovascular disorders such as DCM [37,38]. Although we know from human clinical trials that agents such as TRULI1 and XMU-MP-1 have not been approved yet, CinicalTrials.org does list an approved phase I clinical trial of IK-930 (Oral TEAD Inhibitor Targeting the Hippo Pathway in Subjects With Advanced Solid Tumors - Full Text View - ClinicalTrials.gov). A consequence of the use of anti-cancer agents in patients in need of cardiovascular care has been the emergence of the specialty of Cardio-Oncology [39,40]. Moreover, the search for agents effective in cancer requires consideration of their effects on the heart and blood vessels. A beneficial effect of the pursuit of anti-cancer agents affecting the Hippo pathway and the potential effects on the cardiovascular system is that such studies will inform mechanisms in the heart as well provide means to develop specificity through in depth understanding of cell specific molecular mechanisms as detailed by Lou et al. [37].
